# Current state of genome-scale modeling in filamentous fungi

**DOI:** 10.1007/s10529-015-1782-8

**Published:** 2015-02-21

**Authors:** Julian Brandl, Mikael R. Andersen

**Affiliations:** Department of Systems Biology, Technical University of Denmark, Søltofts Plads 223, 2800 Kongens Lyngby, Denmark

**Keywords:** Filamentous fungi, Genome-scale models, Metabolic engineering, Metabolism, Systems biology

## Abstract

The group of filamentous fungi contains important species used in industrial biotechnology for acid, antibiotics and enzyme production. Their unique lifestyle turns these organisms into a valuable genetic reservoir of new natural products and biomass degrading enzymes that has not been used to full capacity. One of the major bottlenecks in the development of new strains into viable industrial hosts is the alteration of the metabolism towards optimal production. Genome-scale models promise a reduction in the time needed for metabolic engineering by predicting the most potent targets in silico before testing them in vivo. The increasing availability of high quality models and molecular biological tools for manipulating filamentous fungi renders the model-guided engineering of these fungal factories possible with comprehensive metabolic networks. A typical fungal model contains on average 1138 unique metabolic reactions and 1050 ORFs, making them a vast knowledge-base of fungal metabolism. In the present review we focus on the current state as well as potential future applications of genome-scale models in filamentous fungi.

## Introduction

Filamentous fungi have been used for decades in industrial biotechnology exploiting their ability to utilize various sources of nutrients and tolerating adverse growth conditions. For example, tolerance of low pH and the endogenous property of producing citric acid in high amounts have led to the establishment of *Aspergillus niger* as the major source of citric acid production. Furthermore, reflecting the saprobic lifestyle of many filamentous fungi, they harbor a great variety of biomass-degrading enzymes natively produced in high amounts. Additionally, the large diversity of bioactive compounds produced by filamentous fungi is just being recognized as a valuable reservoir of promising new natural compounds. Exploration of the biosynthetic capabilities of these organisms has been facilitated by the availability of genome sequences, thereby enabling the discovery of secondary metabolite clusters being inactive under standard laboratory conditions.

A key requirement for the transition of a new compound into a viable commercial product is the availability of a host producing the compound in sufficiently high amounts. As the organisms are, in general, not evolutionarily optimized to produce a single compound in optimal amounts, process optimizations as well as genetic modifications have to be performed. The rational approach of modifying the metabolism of an organism in order to improve product output constitutes the field of metabolic engineering. Due to the lack of information in the pre-genomic era, the scope of metabolic engineering has been limited to individual pathways not considering inherent interdependencies in the metabolic network. The availability of genome sequence information provides the opportunity to expand the scope of metabolic engineering to the whole metabolism transforming the field towards systems biotechnology. As the process of metabolic engineering represents a bottleneck in the development of many cell factories, accurate predictions by metabolic models could help to reduce the time and costs involved by guiding the efforts towards the most promising set of modifications.

**Fig. 1:**
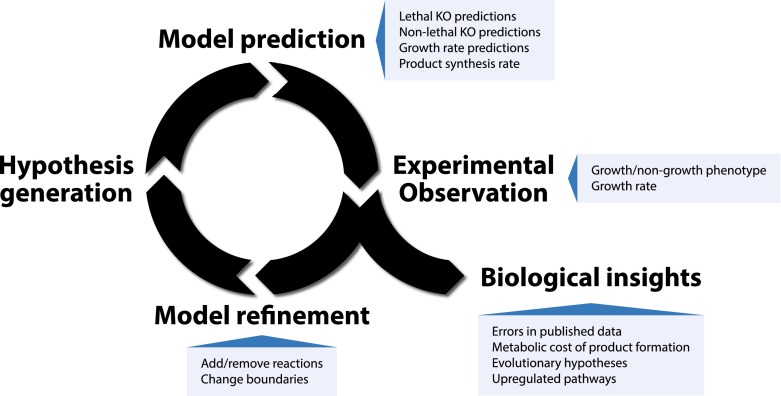
Iterative cycle of model establishment. Examples are taken from Andersen et al. (2009); Melzer et al. (2009); Dreyfuss et al. (2013) and Ledesma-Amaro et al. (2014)

Since the first publication of a complete genome sequence for *Haemophilus influenza* 20 years ago (Fleischmann et al. 1995), the number of published genomes has grown rapidly. The availability of these genomes enabled early reconstruction of the metabolic networks of several species in the groups of viruses (Edwards and Palsson 1999), bacteria (Edwards and Palsson 2000), and yeast (Förster et al. 2003) on the genome scale. These initial drafts have been continuously updated and curated over time, extending the scope and biochemical information contained (Orth et al. 2011; Osterlund et al. 2012). This long period of development demonstrates the iterative nature of model establishment in systems biology where new information is successively included and predictions are validated using experimental data. The process can be visualized as an iterative cycle (see Fig. 1) where the repeated comparison of model predictions with experimental observations leads to biological insights and refinement of the model.

The development of genome-scale network reconstructions (GENRE) in filamentous fungi started considerably later with the first genome being published in 2003 for *Neurospora crassa* (Galagan et al. 2003) followed by the *Aspergillus* *nidulans* (Galagan et al. 2005), *A. oryzae* (Machida et al. 2005) and *A..* *fumigatus* (Nierman et al. 2005) in 2005. To date genome-scale reconstructions for the species shown in Table 1 have been published on the basis of available genome sequences and extensive biochemical legacy information.

**Table 1:** Genome-scale reconstructions of filamentous fungi

Organism	Year	Unique reactions	ORFs	Validation	Reference
*Ashbya gossypii*	2014	1686^b^	506	None	Pitkänen et al. (2014)
	2014	1596	766	Growth predictions Riboflavin production	Ledesma-Amaro et al. (2014)
*Aspergillus clavatus*	2014	2118^b^	695	None	Pitkänen et al. (2014)
*Aspergillus fumigatus*	2014	2330^b^	764	None	Pitkänen et al. (2014)
*Aspergillus nidulans*	2008	676	666	Growth predictions	David et al. (2008)
	2014	2226^b^	745	None	Pitkänen et al. (2014)
*Aspergillus niger*	2008	1190	871	Yield prediction Physiology prediction	Andersen et al. (2008)
	2014	2249^b^	751	None	Pitkänen et al. (2014)
*Aspergillus oryzae*	2008	1053	1314	Growth predictions	Vongsangnak et al. (2008)
	2014	2453^b^	820	None	Pitkänen et al. (2014)
*Aspergillus terreus*	2013	1357	1454	Growth predictions	Liu et al. (2013)
	2014	2401^b^	794	None	Pitkänen et al. (2014)
*Batrachochytrium dendrobatidis*	2014	1979^b^	556	None	Pitkänen et al. (2014)
*Botrytis cinerea*	2014	2173^b^	691	None	Pitkänen et al. (2014)
*Chaetomium globosum*	2014	1930^b^	610	None	Pitkänen et al. (2014)
*Coprinus cinereus*	2014	2080^b^	636	None	Pitkänen et al. (2014)
*Encephalitozoon cuniculi*	2014	536^b^	161	None	Pitkänen et al. (2014)
*Fusarium graminearum*	2014	2182^b^	729	None	Pitkänen et al. (2014)
*Fusarium oxysporum*	2014	2346^b^	786	None	Pitkänen et al. (2014)
*Fusarium verticillioides*	2014	2361^b^	801	None	Pitkänen et al. (2014)
*Laccaria bicolor*	2014	2162^b^	666	None	Pitkänen et al. (2014)
*Magnaporthe grisea*	2014	2152^b^	686	None	Pitkänen et al. (2014)
*Mortierella alpina*	2013	1205	1042	None	Vongsangnak et al. (2013)
*Mucor circinelloides*	2013	1111	1208	None	Vongsangnak et al. (2013)
*Mycosphaerella graminicola*	2014	2363^b^	773	None	Pitkänen et al. (2014)
*Nectria haematococca*	2014	2273^b^	783	None	Pitkänen et al. (2014)
*Neosartorya fischeri*	2014	2200^b^	724	None	Pitkänen et al. (2014)
*Neurospora crassa*	2013	1027	836	Growth predictions Gene essentiality Nutrient rescue Synthetic lethals	Dreyfuss et al. (2013)
	2014	2189^b^	691	None	Pitkänen et al. (2014)
*Penicillium chrysogenum*	2013	1471	1006	None	Agren et al. (2013)
*Phaeosphaeria nodorum*	2014	2449^b^	798	None	Pitkänen et al. (2014)
*Phanerochaete chrysosporium*	2014	2107^b^	655	None	Pitkänen et al. (2014)
*Phycomyces blakesleeanus*	2014	2242^b^	688	None	Pitkänen et al. (2014)
*Postia placenta*	2014	2047^b^	647	None	Pitkänen et al. (2014)
*Puccinia graminis*	2014	1946^b^	564	None	Pitkänen et al. (2014)
*Rhizopus oryzae*	2014	2124^b^	654	None	Pitkänen et al. (2014)
*Sclerotinia sclerotiorum*	2014	2040^b^	653	None	Pitkänen et al. (2014)
*Trichoderma reesei*	2014	2145^b^	697	None	Pitkänen et al. (2014)
*Ustilago maydis*	2014	2068^b^	621	None	Pitkänen et al. (2014)
Summary^a^		1912 (1136)	756 1050)		

^a^Numbers are averages. Numbers in parenthesis is the average without including the CoReCo-models

^b^CoReCo-models; Total number of reactions containing dead-ends

These reconstructions differ considerably in their content of legacy information included, reflecting different strategies of model establishment. The process of manual reconstruction tends to be laborious, as a maximum amount of information is considered leading to the generation of a structured knowledge base. The complementary approach aims at establishing genome-scale reconstructions (semi-)automatically based on sequence comparisons and gene assignments, enabling the prediction of genome-scale networks for less covered species. While these models generally move towards a larger number of genes and reactions included as models are progressively improved, a qualitative comparison with respect to the numbers of reactions and genes included in the resulting models of these different strategies is not directly possible. The majority of automatically generated models contain dead-ends and/or unconnected reactions that have been removed from manually created and curated models, resulting in higher number of genes included. The results from both of these approaches are fragmentary summaries of the metabolic capacities of the organisms requiring additional curation in the form of gap-filling in order to make computational analysis of the networks feasible. The mathematical background of the underlying modeling approach has been reviewed elsewhere (Llaneras and Picó 2008).

Over the last decade, the conceptual foundations for genome-scale metabolic modelling have been laid by the development of standards for model generation (Le Novère et al. 2005; Thiele and Palsson 2010), model exchange (Hucka et al. 2003) and a variety of computational methods for the analysis of the resulting models (Schellenberger et al. 2011). The labor-intensive nature of model construction led to the development of methods for automating a subset of steps in this process (Agren et al. 2013) up to a fully automated generation of genome-scale metabolic reconstructions (Henry et al. 2010; Pitkänen et al. 2014). This long history of methodical developments now enables researchers to easily generate a genome-scale model for their species of interest and subsequently use it for model-driven discovery and rational strain engineering. The availability of genome sequences as well as of established and well-curated models for many species further facilitates the establishment of new genome-scale reconstructions. As filamentous fungi provide a valuable source of new natural products, further increase in the use of genome-scale models in the engineering of these organisms can be expected. In this review we focus on the current state of genome-scale modeling and applications in filamentous fungi and possible future applications.

The applications that have been recorded in filamentous fungi can be divided into three groups. One of the most popular uses for the genome-sale models exploits the underlying collection of information in order to identify gaps in the genome annotation, transfer knowledge to related organisms or generate reduced models for specific analyses. The second category, called model-aided discovery, contains examples where discrepancies between model simulations and experimental data led to the discovery of new biological traits. In the third category, we focus on the applications of genome-scale models for the interpretation of experimental data. This use and the synergism between genome-scale models and omics technologies have been reviewed by Hyduke et al. (2013).

## Structural knowledge base of fungal metabolism

Well-curated genome-scale metabolic models represent a structured knowledge base on the metabolism of the particular organism. The link between available genetic, metabolic and bibliomic information represents a valuable resource for researchers that has not been fully exploited. Its potential applications include development of new genome-scale reconstructions, estimation of network capabilities, as well as analysis of different types of omics data and annotation of related organisms. The enhancement of genome annotations using metabolic networks to represent the relationships between individual enzymatic genes has been coined as “2D annotation” (Reed et al. 2006). The usefulness of this resulting mapping between genes and metabolic functions for the gene function assignment in related organisms has been demonstrated by Liu et al. (2012). The authors used existing genome-scale models as template framework for mapping genes of *Scheffersomyces stipitis.* Similarly, the *Aspergillus niger* genome-scale model (GEM) has been used to assign genes to metabolic pathways in different strains (Andersen et al. 2011).

The existing genome-scale reconstructions for a wide variety of organisms facilitate the development of new metabolic networks as reconstructions of related organisms can be used as a template. This tendency of reusing existing networks is demonstrated by the different network reconstructions of *A. niger,* which have been used as a starting point for the development of the GENREs of *P. chrysogenum* (Agren et al. 2013), *Trichoderma reseii* (Arvas et al. 2011), *Mortierella alpina* and *Mucor circinelloides* (Vongsangnak et al. 2013). Besides this usage for the development of additional genome-scale networks, existing GENREs are actively used as a source to generate reduced models containing all relevant features while facilitating computational analysis. These reduced models can be employed to calculate metabolic fluxes from experimental data as shown by Panagiotou et al. (2008) in their study on the overexpression of phosphoketolase, as well as in the follow-up study on 6-methylsalicylic acid production in *A. nidulans* (Panagiotou et al. 2009).

As genome-scale reconstructions include all theoretically possible metabolic states of an organism, simulating the flux distribution might generate unrealistic results due to the use of otherwise inactive reactions. One example of this is the inability of standard models to accurately predict the secondary metabolite profile produced by a given *Aspergillus* strain. This is partially due to transcriptional and epigenetic regulation of the corresponding gene clusters under standard laboratory conditions (Sanchez et al. 2012). One strategy of dealing with this kind of phenotypic flexibility is the use of omics data as surrogate to determine active reactions in a given environment.

Besides making use of omics data for metabolic modeling, genome-scale reconstructions can be used for the interpretation of omics data by providing the biological framework. There are different examples where genome-scale networks have been used for the analysis of omics data in filamentous fungi. By correlating transcriptional data with calculated flux values during malate production in *A. oryzae* under nitrogen-starvation conditions, Knuf et al. (2013) were able to identify candidates of transcriptionally regulated fluxes representing potential targets for metabolic engineering. Using a reduced model of the *A. niger* GEM, Melzer et al. (2009)developed a similar approach, where they correlated the fluxes through individual pathways with fructofuranosidase production. Using this strategy, they proposed targets for overexpression and gene deletion that would result in an increased fructofuranosidase production.

## Model-aided discovery

The potential of GENREs for hypothesis generation starts already at the stage of building the model when a complete list of reactions present in the organism is compiled. This prediction of missing steps in the metabolic pathways can guide experiments in order to check the predicted function of a gene as well as suggesting functional annotation for orphan genes. This strategy of guiding experimental research using models leads to an iterative cycle refining both the biological knowledge as well as the model itself (see Fig. 1). This use of GEMs for the generation of biological insights is demonstrated by the validation process used in the establishment of the *Neurospora crassa* model (Dreyfuss et al. 2013). Using their reconstruction the authors were able to predict the gene essentially of reactions included in the model with a sensitivity and specificity of 99.1 and 93.6 % respectively. Discrepancy of the predictions with experimental data for the Δ*erg*-*14* mutant led to the discovery of an error in the published knockout strain.

Due to the incomplete characterization of the metabolism for most species, even the most comprehensive of genome-scale reconstructions contain gaps resulting from metabolic reactions not directly characterized in the given organism. However the presence of some specific metabolic reactions can be deduced from observed metabolic capacities of the organism. Identification of these gaps in the network provides the opportunity for guiding the discovery of new genes encoding the enzymatic activity. Candidate genes for individual reactions can be predicted from known homologues in closely related organisms and subsequently tested thereby improving the genome annotation. This kind of strategy has been used by Vongsangnak et al. (2008) to revise the annotation of the *A. oryzae* genome. The authors were able to assign putative functions to 398 newly predicted genes based on gaps identified during the reconstruction of the metabolic network. Similarly, missing reactions in the set-up of the *A. nidulans* GENRE (David et al. 2008) led to the assignment of putative functions to 472 genes based on similarity to orthologous genes catalyzing the missing reaction. This concept of knowledge-driven gene assignment complements the activity of classical gene function prediction strategies based on sequence motifs. During the generation of genome-scale models, gaps in the set of gene assignments are easily detectable, either by manual curation, examining network connectivity, or modeling (Thiele and Palsson 2010).

## Genome-scale model (GEM) aided assessment of phenotypes

Predicting experimental observations not only gives a hint about the outcome of the experiment, but can also give a mechanistic explanation for the observed phenotype. In some of our previous work, we used this approach in the comparison of the citric-acid-production strain *A. niger* ATCC 1015 with the *A. niger* CBS 513.88 used for industrial enzyme production (Andersen et al. 2011). We found an increased expression of genes in the biosynthetic pathways of threonine, serine and tryptophan in the CBS 513.88 strain, reflecting the over-representation of these amino acids in glucoamylase A. Simulation of the fluxes using the GEM of *A. niger* showed that the fluxes carried by these synthetic pathways are required to be at least twice the value in ATCC 1015 in order to support the enzyme production observed in CBS 513.88. This kind of metabolic cost estimation has been proven useful in elucidating the possible reason for the sequence of different acids produced by *A. niger* depending on the ambient pH (Andersen et al. 2009). Considering the acidification potential of the single acids, we have been able to show that the sequence of production is in accordance with an optimal acidification of the surrounding environment at any pH level. This finding suggested that this acidification potential of *A. niger* evolved as a strategy to sustain in a competitive environment.

A similar approach of metabolic cost calculation has been applied to the genome scale model of *A. niger* in order to estimate the metabolic cost associated with by-product formation and reconsumption as shown by (Pedersen et al. 2012). Depending on the magnitude of product formation these kind of metabolic costs are reflected by observable flux changes as demonstrated by Driouch et al. (2012). The authors used the GEM of *A. niger* to determine differences in flux distributions between the wild-type SKANip8 and the fructofuranosidase producing SKAn1015 strain. Using this approach they showed that expression of the *suc1* gene was sufficient to induce redistribution of fluxes favoring the production of fructofuranosidase. Simulation of the flux capacities predicted the possibility of further increasing fructofuranosidase production up to eight-fold without affecting growth performance.

As data-heavy omics-techniques are becoming increasingly important, the need for tools facilitating the interpretation of the numbers generated from these experiments emerges. Genome-scale models have been used as basis for the construction of organism-specific metabolic maps. Utilizing these maps to plot changes in expression profiles from microarray data provides an intuitive access to the data, as significant changes are visualized and put into the metabolic context. The described approach has been used by Salazar and co-workers in their genome-wide study on glycerol metabolism to analyze changes in the expression of metabolic genes between glycerol and glucose fermentation (Salazar et al. 2009). This strategy allows the immediate visual identification of sub pathways showing increased or decreased activity while comparing different conditions. A similar function is now available in the KEGG database (Kanehisa et al. 2014), where omics data can be loaded and plotted onto the corresponding metabolic network. Tools exploiting the topological information contained in these genome-scale reconstructions have been developed for the identification of highly regulated metabolites in transcriptomic data (Patil and Nielsen 2005).

## Discussion

While genome-scale metabolic modeling in *Escherichia coli* and *Saccharomyces cerevisiae* has demonstrated great success in the development and usage of GEMs, the transfer and application to filamentous fungi still lags behind. This delay is not only attributed to the later publication of genome sequences for filamentous fungi, but also reflects the differences in complexity (in particular the number of secreted products and growth morphology) and level of characterization of the individual organisms. A key aspect of metabolism in eukaryotes is the separation of metabolic reactions in different compartments. The correct assignment of metabolic enzymes to these compartments requires detailed information not readily available for a number of components in filamentous fungi metabolism. Even though this imprecision can limit the accuracy of predictions by these models, they still provide a valuable tool for data interpretation besides being a collection of information that is easily accessible. The unique feature of genome-scale network reconstructions is the integration of different types of information from various sources including databases such as KEGG and Swissprot as well as primary literature data. This ordered collection of metabolic reactions is curated with genomic information and literature citations which results in a structured knowledgebase for the specific organism. While organism specific databases pursue a similar goal, the strength of genome-scale reconstructions is the easy accessibility for computational analysis.

Although genome-scale models have been developed for various filamentous fungi (see Table 1), the directly modeling-driven applications have been limited to date compared to other organisms, in particular considering the large industrial application of these organisms. Possibly due to the complexity of fungi, most published examples focus on applying the models for data interpretation and theoretical calculations. However, these retrospective uses have been relatively successful in providing valuable insights in underlying mechanisms and quantitative assessment of the metabolic state causing a specific phenotype. The potential of these models for metabolic engineering, in contrast, has yet to be demonstrated as most of the published efforts end with the prediction of targets, thereby leaving the proof of feasibility to be made. This lacking verification of model predictions might be attributed to the absence of efficient tools for genetic manipulations. Such tools have long been available in *E. coli* and *S. cerevisiae*, enabling multiple genetic modifications in the same host, but have only recently become available in filamentous fungi (Oakley et al. 2012; Jørgensen et al. 2014; Delmas et al. 2014).

Additional factors potentially hampering the final development of these models into predictive tools for genetic modifications are the low percentage of experimentally verified gene assignments, unknown subcellular localization of the different pathways, as well as insufficient data for model validation. Due to advancements in the field of high throughput omics-technologies this necessary data for modeling is becoming more and more available facilitating the prospective use of genome-scale models as well as the model building process. The extension of the knowledge being available for incorporation will furthermore improve the prediction accuracy of existing models rendering more advanced applications possible. This improvement of prediction accuracy over time has already been demonstrated by the genome-scale model of *E. coli* where accuracies of >90 and 80 % have been achieved for single- and double gene knockouts respectively (Monk and Palsson 2014). Recent attempts in the prediction of targets for metabolic engineering mainly consisted of finding suitable knock-out and overexpression targets, leaving a gap between the available molecular biological tools and the set of simulated modifications. However it can be expected that with the advent of synthetic biology, the repertoire of modifications will be extended to the introduction of exogenous metabolic reactions and new regulatory circuits further optimizing the network. The increase in accuracy will also provide the possibility to systematically simulate combinations of modifications leading to the assumption free identification of optimal targets for metabolic engineering.

Continuous extension of the models towards more detailed representations while improving their predictive power demonstrates the iterative nature of model development. The availability of predictive models for important organisms used in industrial biotechnology will provide the opportunity to choose the best performing host platform for a new biotechnological process by simulation, as optimal production of an individual compound requires a very specific metabolic performance. This comparability of genome-scale reconstructions between different species has been severely hampered by the non-standardized modeling practice of the different research groups in the past. With the development of modeling standards (Le Novère et al. 2005), a universal data format for model exchange (Hucka et al. 2003) and repositories for existing models (Schellenberger et al. 2010; Henry et al. 2010) as well as naming ambiguities (Bernard et al. 2014), this approach becomes increasingly feasible.

During the past years, life sciences and biotechnology has begun the transformation from data-poor to a data-rich discipline introducing the need for new tools of analysis and interpretation of the generated data. Genome-scale metabolic networks have been proven useful in contextualizing and analyzing the data acquired by the different omics-techniques. At the same time, the increase in availability and quality of omics data pave the way for the generation of more detailed and context-specific metabolic networks based on the presence of individual metabolic activities. This usage of omics data as a surrogate for the customization of genome-scale models will increase in importance as the sensitivity of the omics technologies is improving. The combination of multi-omics measurements with metabolic modeling provides a powerful tool for the detection and elucidation of unknown relations and interactions. Improvements in the molecular toolbox for genetic engineering will allow for a faster realization of model predictions in the future thereby increasing the speed of model improvement. Taken together the recent developments in the metabolic modeling as well as experimental techniques for generating relevant data are promising for an increased use of model-driven decision making in future.
